# Prevalence of Sarcopenia in Older Patients in Rehabilitation Wards

**DOI:** 10.3390/jpm13060960

**Published:** 2023-06-07

**Authors:** Luigia Brugliera, Alessandra Giordani, Giuseppe D’Angelo, Caterina Trimarchi, Giulia Villa, Tao-Yu Yen, Francesco Bosica, Lorenzo Malatino, David Zweiker, Alessandra Negro, Federica Alemanno, Sandro Iannaccone

**Affiliations:** 1 Department of Rehabilitation and Functional Recovery, I.R.C.C.S. San Raffaele Scientific Institute, Vita-Salute University and San Raffaele Hospital Milan, 20132 Milan, Italy; brugliera.luigia@hsr.it (L.B.);; 2 Department of Cardiac Electrophysiology and Arrhythmology, I.R.C.C.S. San Raffaele Scientific Institute, Vita-Salute University and San Raffaele Hospital Milan, 20132 Milan, Italybosicafrancesco@gmail.com (F.B.); 3 Center for Nursing Research and Innovation, Faculty of Medicine and Surgery, Vita-Salute San Raffaele University, 20132 Milan, Italy; villa.giulia@hsr.it; 4 Department of Clinical and Experimental Medicine, University of Catania, 95131 Catania, Italy; 5 Department of Cardiology, Medical University of Graz, 8036 Graz, Austria; 6 Department of Cardiology and Intensive Care, Clinic Ottakring, 1160 Vienna, Austria; 7 Head nurse at General Surgery, I.R.C.C.S. San Raffaele Scientific Institute, Vita-Salute University and San Raffaele Hospital Milan, 20132 Milan, Italy; negro.alessandra@hsr.it

**Keywords:** sarcopenia, rehabilitation, malnutrition

## Abstract

The multidisciplinary assessment of hospitalized patients via validated scales and tools has become crucial in the early identification of sarcopenia. The objective of this study was to determine the prevalence of sarcopenia and its related factors in patients aged ≥65 years admitted to the neurological rehabilitation departments of cognitive motor disorders and functional motor rehabilitation at the IRCCS Hospital San Raffaele in Milan. Using the algorithm reported by the European Working Group on Sarcopenia in Older People (EWGSOP2), the prevalence of sarcopenia in patients was investigated from 2019–2020. Definite sarcopenia was detected in 161 of 336 recruited patients (47.9%). Age was significantly higher in sarcopenic patients than in those without sarcopenia (median 81 vs. 79 years, *p* < 0.001) and height, weight, and body mass index were lower (*p* < 0.001 for all). The malnutrition screening test (MUST) was higher but still negative in most sarcopenic patients (47.8% vs. 20.6%, *p* < 0.001). Patients with sarcopenia had significantly reduced life autonomy (by Barthel index, median 55 vs. 60 points, *p* < 0.001) and increased mental impairment (tested by MMSE and MOCA, *p* < 0.005 for both). In conclusion, sarcopenic patients were more cognitively impaired and less autonomous in their daily life, but the majority presented with a negative malnutrition screening test.

## 1. Introduction

The concept of sarcopenia was first introduced by Rosemberg in 1989. Since then, the interest in sarcopenia has grown considerably, leading to a wide range of published research articles, which eventually resulted in the formalization of a sarcopenia definition and diagnosis by the European Working Group on Sarcopenia in Older People (EWGSOP 2) in 2018 [1,2].

People affected by sarcopenia are mostly unaware of their condition in the early phase until the progressive degradation of the skeletal muscle mass and function results in a significant reduction of their autonomy, with the risk of adverse outcomes such as physical disability, poor quality of life, and death.

Sarcopenia is associated with a number of poor prognostic factors, such as an increased risk of hospitalization, increased hospitalization time, worse quality of life, and higher expenditure on national health [3].

A study carried out by Dimori et al. in 2017 on long-term-care institution patients screened for the presence of sarcopenia highlighted how a multidisciplinary intervention consisting of adequate nutritional support together with physical exercise led not only to an improvement in terms of skeletal muscle mass (SSM), but also physical performance and muscle strength [4].

The study of the prevalence of sarcopenia is important to reduce its incidence and prevent sarcopenia-related complications. The aim of this research, conducted in the two-year period of 2019–2020, was to determine the prevalence of sarcopenia and to study sarcopenia-associated risk factors in a cohort of patients aged ≥65 years admitted to the neurological rehabilitation department of cognitive motor disorders and functional motor rehabilitation at the IRCCS San Raffaele Hospital in Milan.

## 2. Materials and Methods

This was a single-center prospective observational study focused on sarcopenia in patients admitted to a rehabilitation ward in the two-year period of January 2019–December 2020.

### 2.1. Recruitment

Patients admitted to the rehabilitation ward of the IRCCS San Raffaele Hospital, Milan, Italy, were assessed for eligibility to participate in this study. Inclusion criteria were age ≥ 65 years, ability to undergo bioimpedance analysis, and signed informed consent.

Diagnosis and treatment of sarcopenia:

Assessment for sarcopenia was performed within 48 h of entering the ward. Sarcopenia was diagnosed following a specific multidisciplinary management protocol in which hospitalized patients were subjected to medical, neuropsychological, physiotherapy, and nutritional evaluations. Patients found to have sarcopenia began an individual rehabilitation program during their hospitalization that, when possible after hospital dismissal, continued at home through the assistance care network. This support included ambulatory physiotherapy and nutritional counselling.

### 2.2. Performance Tests

The multidisciplinary of the different wards involved in this study permitted a thorough overview of the patient’s clinical condition, not only from a nutritional standpoint but also considering other factors that may facilitate the identification of sarcopenic subjects.

**Figure 1:**
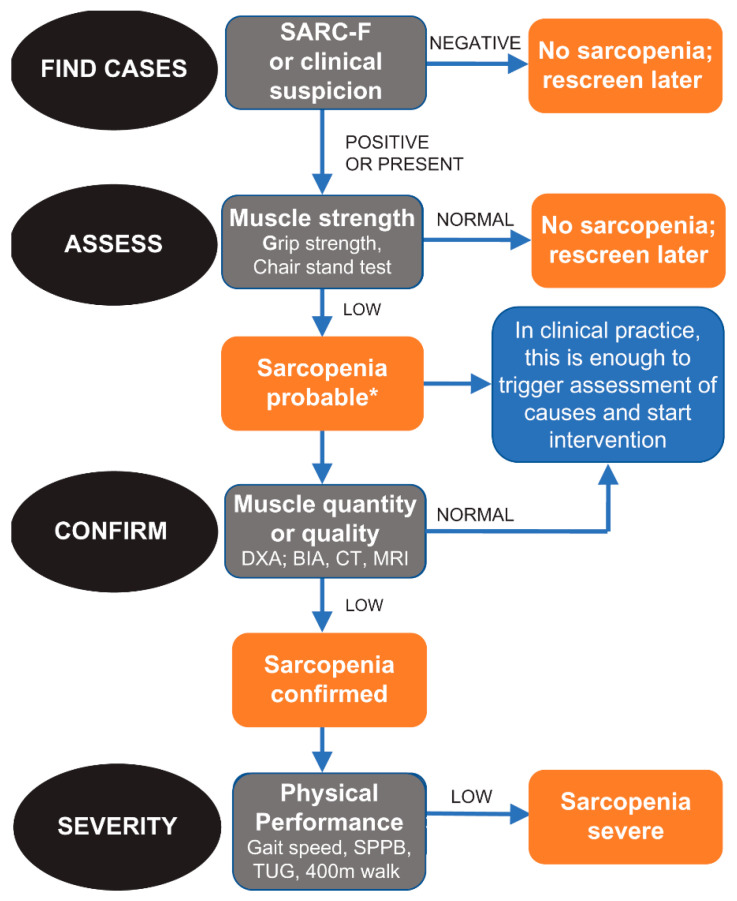
Algorithm developed and suggested by EWGSOP 2.

In this observational study, the algorithm developed and suggested by EWGSOP 2 was used (Figure 1). In fact, this algorithm includes all the necessary characteristics for an appropriate diagnosis of sarcopenia [2]. Specifically, EWGSOP 2 recommends a find–assess–confirm–severity (FACS) pathway to be used in all clinical practices and research studies.

Diagnostic criteria for sarcopenia are the following:

- Low muscle strength;
- Low muscle mass;
- Low physical performance.

A condition of probable sarcopenia (pre-sarcopenia) was identified when the first criteria was met. For a diagnosis of sarcopenia, criteria 1 and 2 had to be met. When all three criteria were met, sarcopenia was defined as severe.

A dynamometer (DynX^®^ Electric hand dynamometer and hand–arm muscle exerciser, MD Systems Inc, Reynoldsburg, OH, USA) was used to measure muscle strength in the upper limbs [5]. The values detected by the measurement of isometric muscle strength using the hand grip were compared with the cut-offs proposed by EWGSOP2 (Table 1) [2].

For the measurement of body composition, and therefore of muscle mass, different instrumental methods can be used. In this study, the bioimpedance analysis, or BIA (using BIA 101 BIVA^®^ PRO, Akern s.r.l., Pisa, Italy), was performed according to the manufacturer’s recommendations. BIA is a noninvasive method that obtains an estimate of the body composition, including the appendicular skeletal muscle mass (ASMM). ASMM indicates the subject’s muscle mass, a parameter that is then compared with the reference cut-offs to complete the diagnosis of sarcopenia (Table 1) [2].

In order to define the severity of sarcopenia, the Short Physical Performance Batter “SPPB” test was used. The test assesses postural balance, walking speed, strength, and endurance. A score below the cut-off determines the severity of the previously detected sarcopenia condition (Table 1) [2]. We further used the SARC-F score to assess its suitability to identify patients at risk of sarcopenia [6].

Furthermore, in addition to biometric measurements, risk scores regarding nutritional status, falling risk, autonomy, and cognitive function were assessed: the Malnutritional Universal Screening Tool (MUST) was designed to identify patients at risk of malnutrition [7]. In this analysis, a cut-off of ≥1 was considered pathological. The risk of falling was assessed with the Conley scale [8] and a value ≥ 2 was considered positive. Life autonomy was estimated with the Barthel index [9], while mental and cognitive functions were analyzed by Mini-Mental State (MMSE) and Montreal Cognitive Assessment (MOCA) [10,11]. We defined no/minimal impairment as MMSE of 24–30 points, moderate impairment in patients with 18–23 points, and severe impairment for remaining patients. Impairment considering MOCA test was defined as a value below 26 points.

### 2.3. Data Collection

A number of parameters, including those relevant for sarcopenia diagnosis, were collected during the multidisciplinary evaluation and recorded in a database (DB). Each enrolled subject agreed to participate in the study by signing an informed consent form. For subjects that were not cognitively competent, the informed consent was obtained from the subject’s legally acceptable representative. This study was approved by the Ethics Committee of the Hospital San Raffaele.

### 2.4. Statistical Analysis

For this study, sarcopenia was the dependent variable, while the independent variables were:

- Demographic data (age; sex);
- Nutritional status and body composition: weight, height, body mass index (BMI), Malnutrition Universal Screening Tool (MUST), appendicular skeletal muscle mass, and Short Physical Performance Batter “SPPB” test;
- The risk of falling (Conley scale);
- Life autonomy (Barthel score);
- Mental and cognitive functions Mini-Mental State (MMSE), Montreal Cognitive Assessment (MOCA).

Descriptive statistics of collected variables were performed as appropriate (median and interquartile range, mean and standard deviation, or frequency and proportion). Normality of distribution was assessed through one-sample Kolmogorov–Smirnov test and Shapiro–Wilk test. To evaluate the association between variables and sarcopenia, nonparametric tests such as the independent sample Kruskal–Wallis test, chi-square test, and Fisher’s test were used. SPSS version 25 (IBM, Armonk, New York, NY, USA) was used for the analysis and graphic presentations.

## 3. Results

A total of 336 hospitalized patients were included in this study, 179 from the Neurological Rehabilitation Department, presenting cognitive motor disorders, and 157 from the Motor Rehabilitation Department. Based on the EWGSOP2 algorithm, 47.9% of the sample (161 out of 336 patients) had a confirmed sarcopenia condition, with significant loss of strength and muscle mass; 30.3% (105 patients) were found to have only loss of strength and therefore showed a condition of probable sarcopenia; and only 70 of the patients (20.8%) did not appear to be sarcopenic.

The population studied comprised 152 men (45.2%) and 184 women (54.8%), with a median age of 79 years (interquartile range, 74–84); age was significantly higher in sarcopenic patients than in remaining patients (median 81 vs. 79 years, *p* < 0.001, Table 2). Median body mass index (BMI) was 24.6 kg/m^2^ and significantly lower in the sarcopenic group (22.2 vs. 26.9, *p* < 0.001). Consequently, underweight patients were overrepresented and overweight patients were underrepresented in this group (Table 2).

SARC-F score was significantly higher in the sarcopenic group, but elevated in the vast majority of the whole cohort. Based on the risk of malnutrition (MUST) performed on the totality of the sample, 66.4% (n = 223) of the subjects showed a low risk of malnutrition (MUST = 0). While the rate of patients with MUST > 0 was significantly higher in the sarcopenic group (47.8% vs. 20.6%, *p* < 0.001), 52.2% of sarcopenic patients still had a physiological MUST score.

Conley and Barthel scales, administered during the nursing assessment, showed that 71.4% (n = 240) were at risk of falling, thus requiring constant attention and observation. A large portion of the sample (43.5%; n = 146) had moderate dependency; whereas only 3.3% (n = 11) of the sample had total independence (score of 100). While a similar proportion of increased Conley scale between both groups was found, sarcopenic patients had a significantly lower Barthel index than remaining patients (median 55 vs. 60, *p* < 0.001).

The neuropsychological evaluation of the cognitive sphere was performed using a series of tests to investigate cognitive impairment. According to the Mini-Mental State Examination (MMSE) scale, only 13.3% (n = 45) of the sample had mild to moderate cognitive impairment. On the other hand, based on the scores of the Montreal Cognitive Assessment (MOCA) scale, a much larger portion of the study sample (47.0%; n = 158) showed cognitive impairment. Both scores were significantly lower in sarcopenic patients (MMSE: 21 vs. 26, *p* < 0.001; MOCA: 15 vs. 19, *p* = 0.001).

## 4. Discussion

This study investigated the prevalence of sarcopenia in hospitalized patients through the use of the up-to-date algorithm suggested by the European Working Group on Sarcopenia in Older People (EWGSOP2). Our results show that approximately half of the patients enrolled (47.9%) had confirmed sarcopenia.

These results underline the high prevalence of sarcopenia among elderly patients admitted to rehabilitation wards and are in line with the results of previous studies conducted in samples of elderly hospitalized patients. Moreover, the current study shows that the incidence of sarcopenia grows with the increasing age of the patents, which is also congruent with previous findings [12,13,14]. We found a significantly higher SARC-F test in the sarcopenic group, but the discriminatory power of the recommended threshold (≥4 vs. 0–3 points) was low.

According to the nutritional assessment, it was possible to evaluate the body mass index and the risk of malnutrition as potential factors for sarcopenia. The results highlight that there is a significant negative association between BMI and the prevalence of sarcopenia. In fact, patients with higher BMI were less likely to suffer from sarcopenia. An interesting explanation is that obese patients may better compensate for high metabolic reserves and resist muscle loss caused by excessive catabolism after an acute event [15]. Still, obesity does not fully prevent sarcopenia, as we found about a quarter of sarcopenic patients (23.6%) to be overweight.

Based on the results of this study, there was a statistical significance between sarcopenia and the risk of malnutrition, but the majority of sarcopenic patients still had a normal MUST score. In this context, the MUST score failed to identify the majority of patients with sarcopenia. Although MUST is a mandatory assessment test in the departments where patients were recruited, it is not sufficient to guarantee correct nutritional therapy for the patient, especially when the patient is affected by sarcopenia. It has to be emphasized that sarcopenia may be present even in patients with normal nutritional status.

Thanks to a multidisciplinary evaluation, which is essential in order to ensure maximum functional recovery, it was possible to investigate aspects other than the nutritional factor and study their association with sarcopenia. For example, a high prevalence of sarcopenic patients were found among those who presented a greater fall risk. In fact, as confirmed by several studies in the literature, the loss of appendicular skeletal muscle mass (ASMM) is strongly associated with the risk for fall [16].

Above all, sarcopenia was more present among those who were less autonomous in daily life activities. This result further supports the usefulness of screening patients upon entrance to the ward. Indeed, we identified the Barthel index on life autonomy as a highly predictive screening test for sarcopenia.

Moreover, the study of the association between cognitive impairment and the presence of sarcopenia could represent a starting point for future investigations. In particular, evidence of the statistically significant correlation between the Mini-Mental State Examination (MMSE) and sarcopenia is relevant, as it is clear that patients who are at a greater risk of presenting sarcopenia are also at greater risk of having a compromised cognitive picture. These results confirm the evidence reported by the meta-analysis of Tao Chun Peng’s group on the correlation between sarcopenia and cognitive impairment [17].

The analysis of these data suggests that the majority of sarcopenic patients tend to have an elevated risk of falling, to present cognitive impairment, and to be less autonomous in their daily life. The coexistence of these conditions can aggravate the state of sarcopenia and the progress of rehabilitation throughout hospitalization.

Our results confirm that sarcopenia can also be associated with other conditions, which outlines a broader picture of frail patients admitted for rehabilitation that deserves to be studied and, above all, detected as soon as possible. Furthermore, the results that emerged in this study are aligned with Rockwood’s conception, which states that sarcopenia must be included in a broad multidimensional model of frailty that also includes psychological and social aspects [18].

The aim of this study was to increase awareness of the high prevalence of sarcopenia. When a frail patient is admitted to the hospital for rehabilitation, it is important for the various medical professionals involved in the patient’s initial evaluation to be aware that not only nutritional and weight-related factors can be interpreted as predictors of sarcopenia. Spreading awareness of the other non-nutritional indicators and implementing a procedure that should certainly include the nutritionist, a figure that is able to manage the nutritional therapy during hospitalization and in the subsequent phases, would make it more likely for the sarcopenia patient to be identified and for the sarcopenic condition to be properly treated.

## 5. Conclusions

In this retrospective analysis of patients admitted to a rehabilitation ward, we found a high proportion of sarcopenic patients. MUST failed to identify the majority of sarcopenic patients. Sarcopenic patients were more cognitively impaired and less autonomous in their daily life. These findings suggest that some variables, which are not necessarily related to nutrition, can be interpreted as predictors of sarcopenia and could facilitate health professionals in identifying the sarcopenic patient.

## Figures and Tables

**Table 1:** Cut-off points for several sarcopenia scores, adapted from Cruz-Jentoft et al. [2].

Test	Cut-off for Men	Cut-off for Women
Handgrip muscle strength (proposed by EWGSOP2)	<27 kg	<16 kg
Appendicular skeletal muscle mass (ASMM)	<20 kg	<15 kg
Short Physical Performance Batter (SPPB)	≤8 point score	

**Table 2:** Baseline characteristics of included patients.

Parameter	Sarcopenic Patients (n = 161)	Non-Sarcopenic Patients (n = 175)	*p* Value
Female gender	59.6% (n = 96)	50.3% (n = 88)	0.100
Age (years)	81 (75–85)	79 (73–82.5)	<0.001
**Nutritional status and body composition**			
Weight (kg)	58 (52–68)	74 (67–84)	<0.001
Height (m)	1.62 (1.55–1.68)	1.66 (1.60–1.72)	<0.001
Body mass index (kg/m^2^)	22.2 (20.1–25.0)	26.9 (23.9–29.7)	<0.001
Overweight (BMI > 25)	23.6% (n = 38)	66.3% (n = 116)	<0.001
Underweight (BMI < 18.5)	14.3% (n = 23)	1.7% (n = 3)	<0.001
MUST > 0	47.8% (n = 77)	20.6% (n = 36)	<0.001
**Sarcopenia evaluation**			
Handgrip (kg)	12.1 (7.4–15.5)	17.8 (11.45–24.1)	<0.001
Reduced handgrip *	100% (n = 161)	60% (n = 105)	<0.001
ASMM (kg)	14.2 (12.6–17.3)	19.6 (16.05–22.58)	<0.001
Reduced ASMM *	100% (n = 161)	12% (n = 21)	<0.001
SPPB	8 (6–8)	7 (5–8)	<0.001
Reduced SPPB *	87.0% (n = 140)	88.6% (n = 155)	0.590
SARC-F score Risk of sarcopenia (≥4) No Risk of sarcopenia (0–3)	8 (6–8) 98.1% (n = 155) 1.9% (n = 3)	7 (5–8) 95.3% (n = 163) 4.7% (n = 8)	<0.001
**Risk of falling**			
Increased CONLEY scale	73.9% (n = 119)	69.1% (n = 121)	0.601
**Life autonomy**			
Barthel index Very dependent (<50) Partially dependent (50–70) Independent (>70) Not assessed	55 (35–65) 42.2% (n = 68) 38.5% (n = 62) 16.1% (n = 26) 3.1% (n = 5)	60 (45–75) 44.6% (n = 78) 28% (n = 49) 21.7% (n = 38) 5.7% (n = 10)	<0.001
**Mental state**			
MMSE No/light impairment (24–30) Moderate impairment (18–23) Severe impairment (0–17)	21 (16.25–26) 43.6% (n = 48) 24.5% (n = 27) 31.8% (n = 35)	26 (22–28) 69.5% (n = 66) 17.9% (n = 17) 12.6% (n = 12)	<0.001
MOCA No Impairment (26–30) Impairment (0–25)	15 (11–20) 2.2% (n = 2) 97.8% (n = 89)	19 (15–22) 7.1% (n = 6) 92.9% (n = 79)	0.001
**Acute disease**			
Neurological disease	55.3% (n = 89)	41.1% (n = 72)	0.012
Orthopedic disease	24.2% (n = 39)	40.6% (n = 71)	0.002
Oncological disease	15.5% (n = 25)	9.1% (n = 16)	0.095
Neurosurgical disease	3.7% (n = 6)	6.3% (n = 11)	0.327
Respiratory disease	0% (n = 0)	1.7% (n = 3)	0.249
Infection	1.2% (n = 2)	0.6% (n = 1)	0.609
Cardiologic disease	0% (n = 0)	0.6% (n = 1)	1.000

* Definitions according to EWGSOP2 in Table 1. MMSE: Mini-Mental State Examination; MOCA: Montreal cognitive assessment test; MUST: Malnutrition universal screening test. Values are expressed as proportion (count), mean or median (interquartile range).

## Data Availability

All underlying data are available from the corresponding author upon reasonable request.
